# T-cell exhaustion in COVID-19: what do we know?

**DOI:** 10.3389/fimmu.2025.1678149

**Published:** 2025-10-29

**Authors:** Roderick Chen-Camaño, Rodrigo DeAntonio, Sandra López-Vergès

**Affiliations:** ^1^ Doctoral Program, Biomedical and Clinical Research Program, School of Medicine – University of Panama, and the Institute of Scientific Research and High Technology Services (INDICASAT-AIP), Panama, Panama; ^2^ Department of Research in Virology and Biotechnology, Gorgas Memorial Institute for Health Studies, Panama, Panama; ^3^ Centro de Vacunación e Investigación (CEVAXIN), Panama, Panama; ^4^ Department of Infectious Diseases, High Complexity Clinical Hospital, City of Health, Social Security System, Panama, Panama; ^5^ Centre for Research, Innovation and Knowledge Management, City of Health, Social Security System, Panama, Panama; ^6^ Sistema Nacional de Investigación (SNI-AIP), Panama, Panama

**Keywords:** COVID-19, SARS-CoV-2, T-lymphocyte exhaustion, T-lymphocyte senescence, CD8-positive T-lymphocytes, immune checkpoint inhibitors, post-acute COVID-19 syndrome

## Abstract

T-cell exhaustion is a terminal state of immune dysfunction characterized by impaired proliferation and effector functions, diminished cytokine secretion, and sustained expression of inhibitory receptors. In coronavirus disease 2019 (COVID-19), increasing evidence links exhausted T-cell phenotypes with poor clinical outcomes, including severe disease, delayed viral clearance, and persistent symptoms associated with Long COVID. Exhaustion results from prolonged antigenic stimulation and inflammatory signals and is marked by transcriptional reprogramming, metabolic and epigenetic dysregulation, and co-expression of inhibitory receptors such as programmed cell death protein-1 (PD-1), T-cell immunoglobulin and mucin-domain containing-3 (TIM-3), and cytotoxic T-lymphocyte-associated protein 4 (CTLA-4). Notably, exhausted phenotypes in COVID-19 frequently coexist with hyperactivation, raising the unresolved question of whether inhibitory receptor expression reflects transient activation or irreversible dysfunction. Emerging therapeutic strategies to reverse these dysfunctional states include immune checkpoint inhibitors, cytokine modulation, metabolic interventions, and epigenetic therapies, although their clinical translation remains at an early stage. Critical research gaps include the scarcity of longitudinal data, incomplete profiling of T-cell subsets across disease stages during COVID-19 and Long COVID-19, and contradictory evidence of vaccine-induced exhaustion with limited understanding of its consequences. This non-systematic literature review synthesizes current advances in COVID-19 immunopathology and therapeutic strategies, underscoring that understanding T-cell exhaustion is crucial to improving outcomes and shaping next-generation immunotherapies and vaccines.

## Introduction

Coronavirus disease 2019 (COVID-19), caused by the novel betacoronavirus severe acute respiratory syndrome coronavirus 2 (SARS-CoV-2), rapidly evolved into a global pandemic, resulting in unprecedented health, social, and economic challenges. While most infected individuals experience mild or asymptomatic disease, a considerable fraction develop severe illness characterized by acute respiratory distress syndrome (ARDS), systemic inflammation, and multiorgan dysfunction (1–3). The clinical and laboratory parameters consistently associated with adverse outcomes include advanced age, comorbidities, elevated levels of inflammatory biomarkers such as interleukin (IL-6) and C-reactive protein (CRP), and profound lymphopenia (4–7). Altogether, these abnormalities signal the central role of immune dysregulation in driving COVID-19 pathogenesis.

Among the adaptive immune components, T lymphocytes play a crucial role in viral clearance and the orchestration of long-term immunity. Both CD4+ helper T cells, which provide essential support for B-cell and innate responses, and CD8+ cytotoxic T lymphocytes (CTLs), which directly eliminate infected cells, are indispensable for protective immunity (8). However, in COVID-19, these responses are frequently compromised, with reduced cell numbers, phenotypic alterations, and functional impairment being consistently observed (9–11).

Severe COVID-19 is characterized by a dysregulated immune landscape marked by hyper-inflammation, aberrant cytokine secretion, and impaired lymphocyte responses (2, 12). High-dimensional immunophenotyping has shown excessive activation of T cells, with increased expression of activation markers such as CD38 and HLA-DR, together with inhibitory receptors, including programmed cell death protein-1 (PD-1) and T-cell immunoglobulin mucin receptor 3 (TIM-3) (4, 9, 13, 14). Importantly, the expression of these molecules does not unequivocally define irreversible exhaustion: several reports indicate that they may also reflect transient hyperactivation during acute infection (15, 16). This overlap underscores the complexity of distinguishing activation from exhaustion and raises critical questions about the stability and reversibility of dysfunctional T-cell states in COVID-19.

The concept of T-cell exhaustion was first described in chronic viral infections such as Human Immunodeficiency Virus (HIV) and the Hepatitis C virus (HCV), where prolonged antigen exposure induces a progressive loss of effector functions and a distinct transcriptional and epigenetic program (17, 18). In COVID-19, similar patterns have been observed but with distinctive features: exhaustion-like signatures can develop rapidly during acute infection, coexist with hyperactivation, and persist into the convalescent phase (4, 9, 16, 19, 20). This distinctive trajectory highlights that T-cell dysfunction in COVID-19 follows patterns unlike those observed in classical chronic viral infections.

The molecular mechanisms underlying dysfunctional T-cell programs include transcriptional regulators such as TOX (Thymocyte selection associated high mobility box) and members of the NR4A (Nuclear receptor subfamily4, group A) family, which cooperate to impose and maintain exhaustion in murine models of chronic viral infection and cancer (21, 22). These molecular pathways contribute to the establishment of a durable epigenetic landscape that renders T cells refractory to subsequent restimulation. While exhaustion-like transcriptional signatures have also been observed in severe COVID-19 (23), it remains unclear to what extent TOX/NR4A programs causally stabilize dysfunction in this acute setting. Notably, TOX has also been described as an extracellular ligand mediating inflammatory activation through the TOX–RAGE (Receptor for advanced glycation end-products) axis in severe pulmonary disease, highlighting context-dependent roles of this factor (24).

COVID-19 therefore provides a unique model system to investigate the boundaries between T-cell activation, dysfunction, and exhaustion, along with their role in both disease pathogenesis and immune memory against re-infections. This complexity underscores the importance of summarizing current knowledge and identifying research gaps in this rapidly evolving field.

## Mechanisms of T-cell exhaustion in SARS-COV-2 infection

T-cell exhaustion represents a multifaceted immunological state shaped by persistent antigen exposure, chronic inflammation, and tissue-specific signals. In the context of SARS-CoV-2 infection, this exhaustion is not only an epiphenomenon of prolonged disease but also a driver of immunopathogenesis (3, 10). Recent studies have characterized the phenotypic, functional, transcriptional, metabolic, and epigenetic alterations that collectively define exhausted T cells during COVID-19 (9, 19, 20, 25, 26). This section dissects the major molecular features of T-cell exhaustion and the mechanistic pathways contributing to immune dysfunction in SARS-CoV-2 infection.

### Hallmarks of T-cell exhaustion

Exhausted T cells are characterized by different dysfunctions, including reduced proliferative capacity, impaired cytokine secretion (notably that of IFN-γ, TNF-α, and IL-2), and compromised cytotoxic granule release (15, 17). T-cell exhaustion during COVID-19 has also been associated with a less effective antiviral response, attenuated cytokine signaling, impaired immune regulation, decreased Th1 polarization, and defective CTL differentiation (19). These functional deficits arise concomitantly with the upregulation of multiple inhibitory receptors, such as PD-1, TIM-3, LAG-3 (Lymphocyte activation gene-3), TIGIT (T-cell immunoreceptor with Ig and ITIM domains), and CTLA-4 (3, 15, 19). In addition, transcriptomic analyses have revealed altered expression patterns consistent with exhaustion, including elevated levels of IFN-γ and ISG15, along with enhanced inflammasome activity. Together, these findings point to a paradoxical phenotype in which T cells appear simultaneously hyperactivated and dysfunctional, particularly in patients with severe disease (19). Importantly, co-expression of these markers is more than phenotypic; it reflects a transcriptionally reprogrammed state reinforced by epigenetic modifications (19).

### Transcriptional regulators as drivers of T-cell exhaustion

Transcriptional regulators, notably TOX and the NR4A family, have emerged as pivotal drivers of the T-cell exhaustion gene program. In models of chronic viral infection and cancer, these factors cooperate downstream of NFAT signaling to impose broad transcriptional and epigenetic changes that suppress effector gene expression and enforce a stable dysfunctional state (21, 22). In the context of COVID-19, single-cell transcriptomic studies have identified exhaustion-associated CD8^+^ T-cell clusters with upregulated TOX expression, particularly in severe disease, suggesting that transcriptional reprogramming contributes to the dysfunctional phenotype observed in these patients (23). Notably, TOX has also been described as an extracellular ligand acting through the TOX–RAGE axis to amplify pulmonary inflammation, highlighting that its role in COVID-19 may extend beyond nuclear transcriptional programming (24).

### Role of inhibitory receptors in COVID-19

The expression of inhibitory receptors is a hallmark of exhausted T cells, with PD-1 (programmed cell death-1) being the most studied (27, 28). Mechanistically, PD-1 signaling disrupts T-cell receptor (TCR) signaling via the recruitment of the SHP-2 phosphatase, which, in turn, inhibits key downstream pathways such as PI3K–Akt and MAPK, both of which are critical for T-cell activation, proliferation, and survival (29, 30). Concurrently, TIM-3 engagement by ligands such as Galectin-9 exacerbates T-cell dysfunction by promoting apoptosis and suppressing effector functions (31, 32). The synergistic impact of these inhibitory pathways leads to profound T-cell impairment, undermining antiviral immunity and enabling persistent viral replication and chronic inflammation (14, 33).

In patients with moderate and severe COVID-19, PD-1+ CD8+ T cells are consistently enriched in peripheral blood and bronchoalveolar lavage fluid. Co-expression with TIM-3, CTLA-4, and TIGIT has been observed in patients requiring intensive care, suggesting a cumulative inhibitory burden that correlates with disease severity (19, 34). Recent studies provide more granularity to the discussion on whether inhibitory receptor expression in COVID-19 reflects true exhaustion or transient hyperactivation. Rha, Jeong (35) demonstrated that PD-1+ SARS-CoV-2-specific CD8+ T cells remained functional during acute infection and contracted during convalescence, consistent with transient activation rather than fixed exhaustion. Wang, Hou (36) reported profound lymphopenia and significantly increased PD-1 expression on CD8+ T cells in severe and critical cases, coinciding with systemic inflammation, suggesting that the upregulation of inhibitory receptors in acute disease may largely represent hyperactivation in the context of severe illness. Bobcakova, Petriskova (13) also found that PD-1 expression was higher in critically ill patients than in moderate cases, while TIM-3 levels did not differ significantly between survivors and non-survivors. Moreover, they reported that at hospital admission, non-survivors displayed higher frequencies of CD8+CD38+ T cells but lower frequencies of CD8+CD38+HLA-DR+ T cells compared to survivors, and the overall activation marker expression declined during recovery. Chattopadhyay, Khare (23) further confirmed this dynamic using single-cell multi-omics, showing that T cells in acute COVID-19 were characterized predominantly by activation signatures, whereas cells from convalescent individuals showed a relative increase in gene expression patterns linked to dysfunctional or exhaustion-like states. Collectively, these findings suggest that exhaustion and hyperactivation are not mutually exclusive but instead represent sequential or overlapping states along a temporal continuum in the immunopathology of COVID-19.

### Cytokine storm and T-cell exhaustion

The cytokine storm associated with COVID-19 is a critical driver of T-cell dysfunction. Elevated levels of IL-6, IL-10, TNF-α, and IFN-γ not only reflect systemic inflammation but also directly contribute to the establishment and maintenance of T-cell exhaustion (2, 12, 37). Of particular interest is IL-10, which exerts immunosuppressive effects by promoting PD-1 expression and suppressing effector gene transcription via STAT3-mediated mechanisms (38, 39). Prolonged exposure to these inflammatory mediators disrupts the metabolic fitness of T cells, alters mitochondrial dynamics, and triggers stress responses that reinforce the exhausted phenotype (40). Importantly, recent studies provide direct evidence that T cells themselves contribute to the inflammatory milieu. For example, CD4+ T cells from patients with severe COVID-19 produce elevated levels of TNF-α, associated with impaired proliferative capacity of SARS-CoV-2-specific CD4+ T cells and increasing their susceptibility to activation-induced cell death. Blockade of the TNF-α pathway restores their normal capacities (41).

Moreover, IFN-γ, although classically antiviral, contributes to tissue damage and T-cell overactivation when persistently elevated (37). This paradoxical role of cytokines exemplifies the delicate balance between protective and pathological immunity in COVID-19.

### Metabolic and epigenetic dysregulation

Metabolic reprogramming serves as both a driver and a consequence of T-cell exhaustion. In COVID-19, exhausted T cells exhibit impaired glycolysis, reduced mitochondrial mass, and altered oxidative phosphorylation, limiting their ability to meet energetic demands during viral clearance (40, 42). These metabolic defects are further compounded by elevated reactive oxygen species (ROS) levels and disrupted fatty acid metabolism, creating a hostile bioenergetic environment that reinforces T-cell dysfunction (40). In addition, exhausted-like T cells display dysregulated NAD+ metabolism, accompanied by elevated adenosine and decreased NAD+ levels in serum from COVID-19 patients (26).

At the epigenetic level, exhaustion is associated with repressive chromatin remodeling. Histone modifications, such as H3K27me3, accumulation at effector gene loci, and DNA methylation at IFNG and PRF1 promoters, lock T cells into an unresponsive state (43). Studies focusing on COVID-19 patients have identified sustained accessibility at exhaustion-related genes (e.g., TOX, Basic Leucine Zipper ATF-Like Transcription Factor (BATF)) and reduced accessibility at effector loci, even in convalescent phases (44–46). This epigenetic rigidity poses a challenge for therapeutic reinvigoration, as checkpoint blockade alone may be insufficient to reverse deeply embedded transcriptional programs in exhausted T cells.

## Clinical implications of T-cell exhaustion in COVID-19

T-cell exhaustion in the context of SARS-CoV-2 infection is not merely an immunological curiosity, it is a clinically actionable phenomenon with profound implications for the disease trajectory, prognosis, and post-acute sequelae (10). The persistence of exhausted phenotypes during acute and convalescent phases of COVID-19 is increasingly being recognized as a central driver of poor outcomes, in terms of both acute respiratory failure and the evolving clinical entity known as Long COVID (10, 15). This section critically analyzes how T-cell exhaustion correlates with disease severity, modulates susceptibility to secondary complications, and contributes to long-term immune dysregulation.

### Impact on disease progression and severity

Numerous studies and meta-analyses conducted since 2022 have robustly demonstrated a link between T-cell exhaustion markers and clinical severity in COVID-19 (3, 10). Patients with moderate to severe disease consistently demonstrate elevated frequencies of PD-1^hi^TIM-3+ CD8+ T cells, reduced absolute lymphocyte counts, and impaired production of IFN-γ and granzyme B—factors intimately tied to effective viral clearance (9, 10, 47). These dysfunctions are not limited to peripheral blood; bronchoalveolar lavage (BAL) samples from ventilated patients show profound T-cell depletion and enrichment of exhaustion markers, suggesting that local tissue environments contribute significantly to immune dysfunction (19). These findings collectively support a model in which both systemic and tissue-specific immune dysregulation underlie severe outcomes in SARS-CoV-2 infection.

Importantly, the degree of exhaustion appears to have predictive value. Studies have shown that increased PD-1 and TIM-3 expression on CD8+ T cells is associated with greater disease severity and poor prognosis (4, 48). Another independent risk factor for 28-day mortality is a significant increase in CD38+HLA-DR+ T cells, a terminally differentiated Treg-like subset exhibiting both activation and exhaustion characteristics (26). These findings have spurred interest in incorporating exhaustion metrics into early triage algorithms to stratify patients based on immune competence.

T-cell exhaustion is a dynamic process. Longitudinal studies have shown that individuals with moderate COVID-19 often exhibit transient exhaustion phenotypes that resolve during convalescence. In contrast, patients with severe or fatal outcomes frequently demonstrate persistent exhaustion signatures, detectable even after viral clearance (2, 48). This prolonged dysfunction suggests that the exhaustion program may become self-sustaining, potentially driven by feedback mechanisms involving chronic inflammatory cytokine exposure, metabolic dysregulation, and altered TCR signaling thresholds. These observations highlight the importance of temporally resolved immune profiling in understanding the trajectory of T-cell dysfunction and its implications for post-acute sequelae of COVID-19.

### Long COVID and T-cell dysfunction

Long COVID, or post-acute sequelae of SARS-CoV-2 infection (PASC), has emerged as a complex clinical syndrome. According to the World Health Organization, this post-COVID-19 condition occurs in individuals with a history of probable or confirmed SARS-CoV-2 infection, usually 3 months from the onset of COVID-19, with symptoms that last for at least 2 months and cannot be explained by an alternative diagnosis (49). Estimates from diverse cohorts suggest that up to 30% of individuals who recover from acute COVID-19 continue to experience symptoms consistent with Long COVID (50). Characterized by symptoms such as fatigue, dyspnea, cognitive impairment (“brain fog”), and myalgia, the syndrome lacks a unifying pathophysiological mechanism. However, persistent immune activation, particularly with features of T-cell exhaustion, is increasingly viewed as a central contributor (51).

Recent studies utilizing high-dimensional flow cytometry and single-cell RNA sequencing (scRNA-seq) have identified the expansion of CD8+PD-1+TOX+ T cells and CD4+ T cells with elevated CTLA-4 expression in Long COVID cohorts up to 12 months post-infection. These populations show reduced proliferative responses to *in vitro* restimulation and diminished IL-2 production—classic markers of exhaustion (10). Importantly, these dysfunctional T cells often coexist with signs of systemic inflammation (e.g., elevated CRP, IL-6), suggesting an ongoing feedforward loop between immune dysregulation and symptom persistence (52).

SARS-CoV-2 infection has been shown to impair both the innate and adaptative immune responses in other tissues, including the oral cavity. This could lead to specific Long COVID syndromes like oral PASC, leading, in turn, to other oral and non-oral pathologies. In the oral cavity, the dysfunction and T-cell exhaustion of different T-cell subsets, including a loss of cytotoxicity, loss of memory T cells, and decrease in Tregs, are associated with risk of mortality, tissue and cell damage, increased levels of autoantibodies with a loss of tolerance, associated with a dysbiotic microbiome, and reductions in tumor surveillance and cytotoxicity (53). Whether these cells are causative or merely reflective of disease remains unclear, but they represent a promising target for interventions aimed at resolving Long COVID symptoms.

Moreover, the chronicity of T-cell exhaustion in PASC raises concerns regarding susceptibility to re-infection and vaccine responsiveness. Several reports have described suboptimal CD8+ T-cell expansion following mRNA booster vaccination in patients with persistent exhaustion, indicating that baseline immune dysfunction may impair vaccine-induced memory formation (54, 55). Understanding the reversibility of this dysfunction is therefore critical, not only for therapeutics but also for shaping future vaccination strategies.

### Susceptibility to opportunistic infections and reactivation events

Beyond compromising antiviral immunity against SARS-CoV-2, T-cell exhaustion contributes to a state of systemic immunosuppression, increasing susceptibility to opportunistic and secondary infections. Notably, the reactivation of latent herpesviruses—including cytomegalovirus (CMV), Epstein–Barr virus (EBV), and herpes simplex virus (HSV)—has been frequently observed in hospitalized COVID-19 patients (56, 57). These events often correlate with elevated expression of PD-1 and TIM-3 on virus-specific T cells, reflecting impaired immune surveillance (51). Such reactivations are clinically significant and have been linked to prolonged hospitalization, increased requirements for mechanical ventilation, and higher mortality rates (58, 59). These findings highlight the broader consequences of T-cell dysfunction in COVID-19 and the need for vigilant monitoring of latent viral reactivation in immunocompromised or critically ill patients.

Nosocomial bacterial and fungal infections, including invasive aspergillosis and candidiasis, are also more frequent among patients with high exhaustion scores (60, 61). The compromised IFN-γ and granzyme B responses in exhausted T cells diminish their capacity to control intracellular pathogens and eliminate infected host cells (62). These data further justify the monitoring of exhaustion markers as part of immune risk profiling in hospitalized patients.

## Therapeutic and immunization strategies to overcome T-cell exhaustion

With the increasing recognition of T-cell exhaustion as a pivotal driver of immune dysfunction in COVID-19, and its impact on clinical severity, considerable attention has turned toward identifying therapeutic strategies that could prevent, mitigate, or reverse this phenomenon. In this section, we explore emerging and repurposed therapeutic strategies under investigation to overcome T-cell exhaustion, including checkpoint blockade, cytokine modulation, metabolic reprogramming, epigenetic interventions, and next-generation vaccine design (**Table 1**).

**Table 1:** Therapeutic strategies to overcome T-cell exhaustion in COVID-19.

Therapeutic strategy	Primary target	Mechanism/effect on T cells	Limitations as treatment	References
Immune checkpoint inhibitors (anti-PD-1, anti-TIGIT, anti-CTLA-4, anti-TIM-3)	Inhibitory receptors (PD-1, TIGIT, TIM-3, CTLA-4)	Partially reverse inhibitory signaling; enhance CD8^+^ T-cell proliferation and IFN-γ production; reduce co-expression of other checkpoints	Potential to exacerbate cytokine storm or autoimmunity; limited clinical data in COVID-19; safety concerns in hyperinflammatory states	(63, 64)
Cytokine modulation (IL-7, anti-IL-10, anti-IL-6; natural compounds such as curcumin, resveratrol)	Cytokine signaling and homeostasis	IL-7: restores T-cell counts and function; IL-10/IL-6 blockade: enhances antiviral T-cell responses but may worsen inflammation; curcumin/resveratrol: modulate TLR–NF-κB signaling, reducing inflammation and exhaustion	Dual effects on inflammation; IL-10 blockade may increase tissue damage; variability in timing and dosing; natural compounds lack standardized dosing or clinical validation	(38, 66, 67, 70, 72)
Therapeutic plasma exchange (TPE)	Plasma cytokines, autoantibodies, inflammatory mediators	Removes pro-inflammatory cytokines and autoantibodies; facilitates immune recovery and antiviral T-cell responses; improves oxygenation and organ failure scores	Limited availability and resource-intensive; unclear contribution of donor plasma components; limited effect on ARDS resolution	(74, 76, 77)
Metabolic reprogramming (metformin, mTOR inhibitors, AMPK activators, mitochondrial antioxidants)	Cellular metabolic pathways (glycolysis, mitochondria, AMPK/mTOR, ROS)	Restores metabolic fitness; enhances proliferation and cytotoxicity; metformin and mTORi modulate bioenergetics; NAD^+^ precursors and antioxidants reduce oxidative stress	Evidence mainly preclinical; long-term metabolic effects unknown; risk of metabolic interference in diabetic or critically ill patients	(40, 42, 79)
Epigenetic modulation (HDAC inhibitors, DNMT inhibitors)	Epigenetic programs (chromatin remodeling, DNA methylation)	Reactivate silenced effector loci; partially restore cytokine secretion and T-cell functions; limitations include toxicity and off-target effects	Potential off-target toxicity; limited *in vivo* validation; risk of global chromatin remodeling	(83, 85–87)
Next-generation vaccine strategies (heterologous prime–boost, multi-epitope, mucosal delivery, optimized adjuvants)	Viral antigens (spike, N, ORF1ab) and adjuvant formulations	Broaden antigenic targets and reduce immunodominance; promote long-lived memory T cells and tissue-resident memory T cells (TRMs); minimize risk of vaccine-induced exhaustion	Still experimental; unclear durability of T-cell protection; potential for immune imprinting	(88, 90, 91, 93)

ICI, immune checkpoint inhibitor; TPE, therapeutic plasma exchange; ARDS, Acute Respiratory Distress Syndrome; HDAC, histone deacetylase; DNMT, DNA methyltransferase; mTOR, mechanistic target of rapamycin; AMPK, AMP-activated protein kinase; ROS, reactive oxygen species; TRM, tissue-resident memory T cell; NF-κB, nuclear factor kappa-light-chain-enhancer of activated B cells.

### Immune checkpoint inhibitors

ICIs such as anti-PD-1 and anti-TIGIT monoclonal antibodies have shown promise in reversing T-cell exhaustion in cancer and chronic viral infections. Therapeutically, targeting these pathways has shown potential for COVID-19 in preclinical models, as multiple *in vitro* and animal studies suggest partial restoration of T-cell function following checkpoint blockade (63). Ex vivo studies using T cells from COVID-19 patients have demonstrated that PD-1 blockade reverses the increase in expression of the positive costimulatory marker OX40 in the CD8+ T cell fraction observed in patients who have recovered from COVID-19. Furthermore, anti-PD-1 treatment results in an increase in the number of IFN-γ-producing cells upon antigenic stimulation when compared to SARS-CoV-2 peptide-only treated cells, along with enhanced proliferative capacity, confirming that PD-1 blockade enhances the specific T-cell immune response to SARS-CoV-2 (63). Notably, other ex vivo studies demonstrated that anti-TIGIT treatment reduces the expression of other checkpoint receptors, including PD-1 and TIM-3, on CD8+ T cells (64). However, caution remains regarding their clinical application in COVID-19 due to safety concerns of exacerbating cytokine storms or triggering autoimmune pathology, particularly in the hyper-inflammatory microenvironment characteristic of severe COVID-19 (65).

### Cytokine modulation

Given the prominent role of cytokine dysregulation in driving T-cell exhaustion, the therapeutic modulation of cytokine pathways represents a compelling strategy. IL-7, a γ-chain cytokine essential for T-cell survival and homeostasis, has shown encouraging results in restoring lymphocyte counts and function in septic patients and was repurposed early in the pandemic (66). Specifically, in a phase 2 trial of 109 patients, IL-7 was well tolerated and associated with a 44% reduction in hospital-acquired infections compared to placebo. While absolute lymphocyte counts increased in both the treatment and control groups, patients receiving IL-7 without concurrent antiviral therapy showed a 43% greater final lymphocyte count (66).

While IL-10 has traditional anti-inflammatory properties, its elevated levels in severe COVID-19 correlate with poor outcomes, probably in response to high inflammation in these patients, and it has been suggested that IL-10 may contribute to T-cell exhaustion (67, 68). Studies in rhesus macaques demonstrated that IL-10 blockade enhances SARS-CoV-2-specific CD4+ and CD8+ T-cell responses while increasing lung inflammation, inflammatory markers, and pulmonary lesions (38). This effect highlights the challenge of targeting IL-10 therapeutically, as its beneficial effect on T cell responses might be counteracted by its detrimental effect on exarcerbating inflammation in severe COVID-19.

IL-6 is associated with cytokine storm and COVID-19 severity; thus, IL-6 blockade was proposed early as a therapeutic tool, with studies reporting conflicting results regarding its benefit. Studies reported that monoclonal tocilizumab had a beneficial effect on all-cause mortality in the short term, but this was not reproducible with other blockades (69, 70). However, a recent randomized double-blinded trial confirmed that IL-6 blockade does not have a strong beneficial effect on patients (71).

The use of natural compounds, such as curcumin and resveratrol, has been proposed as an adjunctive strategy for cytokine modulation in COVID-19. Both agents are reported to suppress TLR–NF-κB signaling, thereby reducing the production of pro-inflammatory cytokines, including TNF-α, IL-1β, and IL-6, which may help to dampen the cytokine storm (72, 73). Beyond these general effects, experimental studies in other viral and inflammatory models suggest that curcumin can also influence regulatory cytokine profiles, whereas resveratrol, through activation of SIRT1, can attenuate NF-κB transcriptional activity and reduce IL-6 and TNF-α secretion. Evidence in COVID-19 patients, however, remains preliminary, and these compounds should be regarded as illustrative examples of how cytokine modulation might be leveraged to protect T-cell function, with the therapeutic benefits depending strongly on the timing and disease context.

### Therapeutic plasma exchange

TPE consists in repeatedly purging a critically ill ICU (Intensive care unit) patient’s plasma with plasma from healthy donors to remove excess inflammatory mediators and pathogenic autoantibodies, in order to attenuate the hyper-inflammatory state and subsequent disturbance of innate and adaptative immunity. This approach also avoids adaptative response inhibition, as compared to therapies such as corticosteroids or JAK inhibitors. Although its effects are modest, TPE has demonstrated significant improvements in oxygenation parameters, multiorgan failure scores, and mortality rates (74–76). A prospective randomized controlled clinical trial with a small number of ICU patients with ARDS showed that TPE, used with standard treatment (including corticosteroids and high-flux-rate oxygen), accelerates immune cell recovery and contributes to the development of appropriate antiviral T-cell responses (lesser cytokine storm, fewer autoantibodies, less hyperactivation, less exhaustion); however, even when it allowed immune recovery, it did not have an effect on ARDS parameters (77).

Future studies are needed to determine whether TPE’s effect is based only on the removal of excess inflammatory cytokines and autoantibodies or if other components of the healthy donor’s plasma (like soluble proteins, extravesicular vesicles, or miRNA) have a role in the immune recovery mechanism. The question of why immune recovery is not enough to significantly improve ARDS is still open, and the timing might be a crucial parameter to take into consideration.

### Metabolic reprogramming

Metabolic dysfunction is a hallmark of exhausted T cells, rendering metabolic reprogramming a novel therapeutic frontier. Exhausted T cells display impaired glycolytic flux, dysfunctional mitochondria, and altered fatty acid metabolism (78). Restoring metabolic fitness can enhance survival, effector function, and cytokine production (1). Metformin, a widely used antidiabetic drug, has shown immune-modulatory effects via AMPK (AMP-activated protein kinase) activation and mitochondrial restoration. Retrospective studies have suggested lower rates of severe disease and mortality among metformin users with COVID-19, though the causality remains under debate (79, 80). More targeted agents such as mTOR inhibitors (mechanistic target of rapamycin inhibitors) or HIF-1α stabilizers are under investigation for their ability to support T-cell bioenergetics and reverse exhaustion-related deficits (81). Additionally, agents that improve mitochondrial biogenesis or limit oxidative stress—such as NAD+ precursors and mitochondrial-targeted antioxidants—are currently being evaluated in animal models and early-phase clinical trials for viral infections, with relevance to COVID-19 and Long COVID (82).

### Epigenetic modulation

T-cell exhaustion is not solely a functional state; it is also epigenetically imprinted. Exhausted T cells exhibit stable chromatin accessibility patterns at exhaustion-related genes and reduced accessibility at effector loci. This repressive epigenetic state is a barrier to complete functional recovery and poses a challenge for therapeutic reinvigoration, as checkpoint blockade alone or even antigen clearance may be insufficient to reverse deeply embedded transcriptional programs (83–85). Targeting this epigenetic rigidity is therefore an emerging therapeutic strategy. Histone deacetylase (HDAC) inhibitors, originally developed for oncology, have demonstrated partial reactivation of effector programs in exhausted T cells in preclinical viral models (86). Similarly, DNA methyltransferase inhibitors have been shown to reverse repression at cytokine gene loci, restoring partial functionality *in vitro* (87). However, translating these findings to COVID-19 faces challenges, including toxicity profiles, systemic off-target effects, and the timing of administration. The development of tissue-specific or T-cell-targeted epigenetic modulators may enhance therapeutic specificity and safety.

### Next-generation vaccine strategies

While current COVID-19 vaccines provide robust protection against severe disease, emerging evidence has highlighted the need to optimize T-cell responses to sustain long-term immunity and mitigate the risk of T-cell exhaustion. To address this, several next-generation vaccine strategies are under investigation. Heterologous prime–boost regimens that combine mRNA and protein subunit platforms aim to enhance antigenic breadth and reduce reliance on a single epitope (88, 89). In parallel, multi-epitope constructs targeting conserved non-spike proteins, such as nucleocapsid and ORF1ab, are being developed to reduce immunodominance and mitigate exhaustion by promoting more balanced T-cell responses (90, 91).

Efforts are also underway to optimize adjuvant formulations that modulate the inflammatory milieu, favoring the generation of long-lived memory T cells rather than terminally exhausted phenotypes (92). Furthermore, mucosal delivery strategies—including intranasal vaccines—are being designed to generate tissue-resident memory T cells at the primary site of viral entry while reducing systemic inflammation that contributes to dysfunction (93).

Importantly, these strategies must also account for age-related immune remodeling, such as immunosenescence in older adults, and baseline immune dysregulation in high-risk populations (e.g., individuals with obesity, diabetes, or chronic cardiovascular disease)—groups that are particularly susceptible to T-cell exhaustion and poor vaccine durability.

Tailoring vaccine platforms and adjuvants to these vulnerable cohorts may be essential to ensure broad protection and sustained immune competence. Collectively, these approaches emphasize that durable vaccine-induced protection will depend not only on the breadth of coverage against variants but also on the capacity to preserve T-cell functionality, reduce exhaustion, and sustain effective memory responses as key hallmarks of next-generation SARS-CoV-2 vaccines.

## Research gaps in T-cell exhaustion and COVID-19

Despite rapid scientific advances in our understanding of T-cell exhaustion in COVID-19, significant knowledge gaps persist. These limitations impede the development of targeted immunotherapies, hinder risk stratification in clinical care, and obscure our comprehension of long-term immune recovery following SARS-CoV-2 infection. This section outlines the most pressing research areas related to the temporal, mechanistic, cellular, and translational aspects of T-cell exhaustion in COVID-19.

### Longitudinal studies on T-cell exhaustion dynamics

A major limitation in the current literature is the scarcity of robust longitudinal studies that systematically follow the initiation, persistence, and resolution of T-cell exhaustion throughout the course of SARS-CoV-2 infection. Most available data are derived from cross-sectional analyses, which provide time-specific snapshots and fail to capture the temporal dynamics of T-cell dysfunction (11). Consequently, critical questions remain unresolved, such as whether T-cell exhaustion in COVID-19 is a transient and reversible adaptation, or whether it gives rise to a durable, non-adaptative state, particularly in individuals with severe or prolonged disease (4, 37, 42, 54). Addressing this knowledge gap is essential for guiding therapeutic strategies aimed at restoring immune competence and preventing long-term immune dysregulation.

Recent attempts to bridge this gap using serial blood sampling and scRNA-seq have demonstrated that exhaustion markers can persist for several months post-recovery, especially in CD8+ T cells (94–96). However, these findings are limited by small cohort sizes, heterogeneous definitions of Long COVID, and variable sampling intervals. There is an urgent need for high-powered prospective studies with standardized assays to longitudinally track exhaustion phenotypes across diverse clinical contexts, different SARS-CoV-2 epidemiological environments, and different demographic groups.

Equally important is the spatial aspect: peripheral blood does not fully reflect the exhaustion states within tissues such as the lung, lymph nodes, and bone marrow. Techniques like fine-needle aspiration and spatial analysis may help elucidate the tissue compartmentalization of exhaustion and clarify how it relates to systemic biomarkers (97, 98).

### Mechanistic insights into exhaustion reversal

The mechanisms of T-cell exhaustion initiation, maintenance, and reversal need to be explored in more detail to design better therapeutic tools. While checkpoint inhibitors have revolutionized cancer immunotherapy, their application in infectious diseases—including COVID-19—remains largely theoretical (**Table 1**). A critical research gap concerns whether the exhausted phenotype observed in SARS-CoV-2 infection is mechanistically and epigenetically reversible and, if so, under what conditions (65).

Recent experimental evidence suggests that PD-1 blockade can effectively reverse T-cell exhaustion and enhance SARS-CoV-2-specific T-cell responses in COVID-19 patients (63). Additionally, combination strategies targeting metabolism (e.g., with mTOR inhibitors or AMPK activators) (65), cytokine signaling (e.g., IL-10 or IL-6 blockade) (38), or epigenetic modifiers (e.g., HDAC inhibitors) (86) have shown potential in preclinical models but remain untested in clinical settings. It is essential to clarify the mechanisms of action, therapeutic windows, and reliable biomarkers of responsiveness for these interventions before they can be translated into clinical practice.

### Role of T-cell subsets and clonotypes in exhaustion susceptibility

The heterogeneity of T cells’ responses to SARS-CoV-2 adds complexity to exhaustion studies. Not all T cells are equally prone to exhaustion: memory precursors, effector subsets, and tissue-resident populations exhibit differential sensitivity to chronic stimulation and inhibitory signaling (16). However, few studies have stratified exhaustion phenotypes by lineage, clonotype, or functional state. Tissue-resident memory T cells (TRMs) in pulmonary tissue display unique exhaustion dynamics, possibly modulated by local cytokines and epithelial interactions (38). Even the clonotype diversity and expression of exhaustion markers differ depending on the T-cell and memory-cell subtypes (25). To advance this field, high-resolution lineage tracing and TCR-seq (T-cell receptor sequencing) approaches must be employed to track the exhaustion trajectories of SARS-CoV-2-specific T-cell clones over time. Such data could inform vaccine design by identifying epitopes that preferentially elicit durable, non-exhausted responses.

### Vaccine-induced T-cell exhaustion and booster optimization

While global attention has largely focused on the humoral immune response elicited by COVID-19 vaccination, cellular immunity (particularly memory CD8^+^ T-cell responses) plays a critical role in long-term protection against severe disease and viral variants.

Recent studies have raised concerns that repeated mRNA vaccine boosting, particularly in individuals with prior SARS-CoV-2 infection or ongoing systemic inflammation, may contribute to the emergence of T-cell exhaustion-like phenotypes (54, 99). Longitudinal data from vaccine cohorts have shown upregulated expression of inhibitory receptors such as PD-1 and TIGIT on SARS-CoV-2-specific CD8^+^ T cells following multiple booster doses, with the effect being more pronounced in older adults and individuals with underlying comorbidities (54). On the other hand, Benoit, Breznik (100) demonstrated that repeated SARS-CoV-2 vaccination was not associated with increased T-cell exhaustion in older frail adults, immunosuppressed individuals, or healthy adults, with no significant changes in combined exhaustion marker expression on spike-specific CD4^+^ T cells following the third or fourth SARS-CoV-2 vaccination.

The relationship between vaccination and T-cell exhaustion appears more nuanced than initially feared. To address these challenges, future studies must define optimal booster intervals, vaccine antigen design, adjuvants, and immunological correlates of durable protection that balance robust memory formation with the minimization of exhaustion risk.

### Lack of translational biomarkers

Finally, a critical translational gap lies in the absence of standardized, clinically actionable biomarkers for T-cell exhaustion. While PD-1, TIM-3, and TOX are informative in research settings, they are rarely integrated into clinical decision-making due to their technical complexity and variability.

A consensus panel of exhaustion markers, validated across cohorts and assay platforms, is needed for implementation in routine diagnostics and clinical trials. Machine learning approaches leveraging transcriptomic, proteomic, and metabolic data have begun to identify exhaustion-related signatures predictive of hospitalization, intensive care unit admission, and mortality (101). However, these models require validation across populations and must account for confounders such as age, sex, comorbidities, and prior vaccination.

## Conclusion

T-cell exhaustion represents a multifactorial phenomenon at the crossroads of immunopathology and disease resolution in SARS-CoV-2 infection. As the pandemic has transitioned into an endemic phase, exhaustion has emerged as a central determinant of both acute severity and persistent immune dysfunction in Long COVID. Research over the past four years has identified its core hallmarks—upregulation of inhibitory receptors, impaired cytokine production, metabolic derangements, and epigenetic remodeling—while also revealing substantial heterogeneity across disease stages, patient populations, and tissue compartments. Clinically, exhaustion not only shapes outcomes during acute infection but also influences vaccine responsiveness and the durability of protective memory. Preventing or reversing exhaustion is therefore critical for improving survival, mitigating post-acute sequelae, and strengthening immunity against emerging variants.

## Future perspectives

Despite these advances, major challenges remain. Methodological gaps persist, including limited longitudinal studies, incomplete profiling of T-cell subsets, and the absence of validated clinical biomarkers. Therapeutic strategies such as those involving checkpoint inhibitors, metabolic enhancers, cytokine modulators, and epigenetic agents are promising but remain experimental and must balance immunity reinvigoration with inflammation control. Looking forward, three priorities are clear: (1) systematically incorporating exhaustion markers into clinical immunophenotyping, particularly in severe and Long COVID; (2) tailoring therapies through patient stratification to identify those most likely to benefit from reinvigoration strategies; and (3) designing next-generation vaccines that optimize not only antibody durability but also exhaustion-resistant T-cell responses. Beyond COVID-19, the rapid onset and persistence of exhaustion observed here challenge paradigms from chronic infection and cancer models, urging a redefinition of how this dysfunctional state is understood and targeted across infectious and non-infectious diseases.
